# Role of Vitamin D Status and Alterations in Gut Microbiota Metabolism in Fibromyalgia-Associated Chronic Inflammatory Pain

**DOI:** 10.3390/biomedicines13010139

**Published:** 2025-01-09

**Authors:** Caterina Saija, Maria Paola Bertuccio, Alberto Scoglio, Vincenzo Macaione, Francesco Cacciola, Giuseppe Micalizzi, Daniela Caccamo, Carolina Muscoli, Monica Currò

**Affiliations:** 1 Department of Biomedical and Dental Sciences and Morpho-Functional Imaging, University of Messina, 98125 Messina, Italy; caterinasaija93@gmail.com (C.S.); mariapaola.bertuccio@unime.it (M.P.B.); alberto97scoglio@gmail.com (A.S.); monica.curro@unime.it (M.C.); 2 Department of Clinical and Experimental Medicine, University of Messina, 98125 Messina, Italy; vincenzo.macaione@unime.it; 3 Messina Institute of Technology, Department of Chemical, Biological, Pharmaceutical and Environmental Sciences, Former Veterinary School, University of Messina, 98168 Messina, Italy; cacciolaf@unime.it (F.C.); giumicalizzi@unime.it (G.M.); 4 Department of Health Sciences, Institute of Research for Food Safety and Health (IRC-FSH), University “Magna Graecia” of Catanzaro, 88100 Catanzaro, Italy; muscoli@unicz.it

**Keywords:** fibromyalgia, chronic inflammatory pain, gut dysbiosis, gut microbiota metabolites, vitamin D, pro-inflammatory cytokines, Kyn/Trp, SCFAs

## Abstract

**Background/Objectives**: Several studies suggest gut microbiota metabolites as important immuno-modulators in inflammatory pain. We aimed to investigate the relationship between vitamin D status and gut dysbiosis markers in fibromyalgia (FM)-associated chronic inflammation. **Methods**: Blood samples were collected from sixty-eight female FM patients (49.9 ± 12.35 years). Pain intensity was assessed by FIQ-R. The serum levels of the pro-inflammatory cytokines TNF-α, IL-1β, IL-6, IL-17, IFN-γ, as well as those of vitamin D (25(OH)D3) and the kynurenine/tryptophan ratio (Kyn/Trp) were determined by ELISA and HPLC, respectively. The plasma levels of the SCFAs acetate, butyrate, and propionate were detected by GC-MS. **Results**: A mean FIQ-R score indicated that the patients could be classified as having moderate FM. The mean levels of all cytokines, but IL-6 and IL-1β, were higher than the normal reference values. The highest concentrations of cytokines were observed in patients showing the highest FIQ-R scores and the lowest 25(OH)D3 levels. Deficient levels of acetate were found paralleled by an increase in Kyn/Trp. The highest acetate concentrations were detected in patients with the lowest FIQ-R scores and 25(OH)D3 levels. Significantly negative correlations were found between 25(OH)D3 concentrations and FIQ-R scores (*p* = 0.007) as well as IL-17 levels (*p* = 0.002) and between acetate and TNF-α (*p* = 0.040) as well as FIQ-R scores (*p* = 0.028), while significantly positive correlations were observed between Kyn/Trp and IL-17 (*p* = 0.027) as well as IFN-γ (*p* = 0.003). **Conclusions**: Our preliminary data suggest that the vitamin D status along with altered gut microbiota metabolism plays a major role in FM-related inflammatory pain. Replication of these findings in a larger cohort is required to provide additional insights.

## 1. Introduction

An alteration in pain processing pathways, leading to widespread chronic pain perception even in the absence of noxious stimuli, has been reported in fibromyalgia (FM), a potentially disabling multifactorial disorder affecting 2–3% of the general population (women/men, 3:1). FM is also characterized by concomitant non-specific symptoms, such as fatigue, irritable bowel syndrome, sleep disturbances, mood disorders, and cognitive dysfunction [1,2]. Low-grade systemic inflammation and neuroinflammation, together with oxidative/nitrosative stress, and expansion of Th1 pro-inflammatory lymphocytes, are common features in FM patients [3,4,5]. Moreover, reduced gut bacteria diversity leading to alterations in gut metabolism were reported, also, in correlation with FM severity [6]. Defects in genes coding for detoxification and antioxidant defense enzymes, neurotransmitters, and cytokines have been suggested as disease susceptibility factors [7,8,9,10,11]. To date, acquired factors contributing to FM development have not yet been elucidated.

Several studies indicate that the gut microbiota may play an important modulatory role in inflammatory pain by interacting with sensory afferent neurons. This interaction can occur either directly, through the secretion of metabolites such as short-chain fatty acids (SCFAs), or indirectly, through first signaling to immune cells [12]. Microbiota-derived mediators also include agonists of Toll-like receptors. These agonists indirectly increase neuronal excitability by triggering the release of pro-inflammatory factors from immune cells, which in turn enhances pain. On the other hand, bile acids can indirectly reduce neuronal excitability by promoting the release of opioids from immune cells, leading to pain relief [3,9,13,14,15,16,17,18,19]. Anyhow, further mechanistic studies are needed to gain a deeper understanding of the crosstalk between the microbiota and sensory neurons in pain processing.

An imbalance in tryptophan (Trp) and its endogenous metabolite kynurenine reflects alterations in gut bacteria diversity/metabolism and strongly affects host immune system–gut bacteria interactions, inducing chronic immune activation, inflammation, and oxidative stress. Metabolomics studies in FM patients showed alterations in SCFAs and Trp levels, correlated with the reported pain severity [20,21,22,23].

An inverse correlation of pain with vitamin D levels has been reported in disorders caused by a dysregulated immune system. Low vitamin D levels were associated with inflammation, impaired endocannabinoid system, and gut dysbiosis in mice, as well as inflammatory changes in gut barrier integrity and bacteria diversity, mediated through vitamin D interaction with gut metabolism in intestinal disorders [24,25,26,27,28,29,30,31,32].

The aim of this study was the evaluation of the role of vitamin D in FM-associated chronic inflammatory pain, focusing on the influence exerted by vitamin D levels on the concentrations of inflammatory markers and gut microbiota metabolites.

## 2. Materials and Methods

### 2.1. Study Cohort

In this study, 68 Caucasian women (49.9 ± 12.35 years) were recruited in collaboration with the Psycho-Neuro-Endocrine-Immunology (PNEI) clinic of the Papardo-Piemonte Hospital of Messina.

The inclusion criteria for the study population were a previous fibromyalgia diagnosis according to the criteria of the American College of Rheumatology (ACR) for this disorder [2,33] and a pain score ≥ 4 on the Fibromyalgia Impact Questionnaire Revised (FIQ-R) pain item at screening.

The exclusion criteria were non-FM pain (including diabetic peripheral neuropathy, post-therapeutic neuralgia, traumatic injury, prior surgery, or complex regional pain syndrome), infectious arthritis, autoimmune disease, or other widespread rheumatic diseases [2,33].

Fasting blood samples were collected from all participants at the Unit of Clinical Biochemistry of the Polyclinic Hospital University “G. Martino”, using tubes with EDTA or clotting activator and separator gel. Plasma and serum were obtained after blood centrifugation, and aliquots were stored at −20 °C until analysis.

Pain intensity in FM patients was assessed by the Fibromyalgia Impact Questionnaire Revised (FIQ-R) [7].

All participants released an informed written consent. This study protocol was approved by the local Ethics Committee of the Polyclinic University of Messina, Italy (protocol n. 24-22, date approval 15 February 2022) and conducted in accordance with the amended Helsinki declaration.

### 2.2. Determination of Vitamin D

The quantitative determination of vitamin D (25(OH)D3) levels was performed in serum by high-performance liquid chromatography (HPLC) using commercially available kits (Recipe, Munich, Germany), according to the manufacturer’s instructions, and an Agilent 1200 Series HPLC system (Agilent Technologies Italia, Cernusco sul Naviglio, Milan, Italy) [30].

### 2.3. Determination of Tryptophan and Kynurenine

For the deproteinization, 200 µL of sample was mixed with 800 µL of methanol, incubated for 20 min on ice, and finally centrifuged at 13,000 RPM for 10 min at 0 °C. Then, 40 µL of supernatant was analyzed using an UltiMate 3000 UHPLC System (Thermo Fisher Scientific, Waltham, MA, USA) equipped with a multiwavelength UV detector, controlled by Chromaleon 7 Chromatography Data System (CDS) software. Isocratic separations were run on a Waters column (300 × 3.9 mm) with distilled water/acetonitrile (40:60 *v*/*v*) at a flow rate of 1.0 mL/min. Injections of 20 μL for each biological sample were performed. Elution was monitored by a UV detector at 280 nm for tryptophan and 360 nm for kynurenine.

The calibration curves for each analyte were obtained by injecting various amounts of standards (Sigma-Aldrich, Darmstadt, Germany) into the HPLC system and used as reference calibration curves for analyte quantification in samples.

### 2.4. Determination of Pro-Inflammatory Cytokines

The quantification of the serum levels of the pro-inflammatory cytokines tumor necrosis factor-alpha (TNF-α), interleukin-beta (IL-1β), interleukin-6 (IL-6), interleukin-17 (IL-17), and interferon-gamma (IFN-γ) was carried out by commercially available ELISA kits (TNF-α, IL-1β, IL-6: MyBioSource, San Diego, CA, USA; IL-17, IFN-γ: Sigma-Aldrich, Milan, Italy), according to the manufacturer’s instructions.

The absorbance was determined at 450 nm using a microplate reader (Tecan, Milan, Italy).

### 2.5. Determination of Advanced Oxidation Protein Products

The serum concentrations of advanced oxidation protein products (AOPPs) were assessed by a colorimetric assay using chloramine-T (Sigma-Aldrich, Missouri, USA) as a standard, as previously reported [34]. Chloramine-T absorbance at 340 nm was linear within the range of 0–100 µmol/L. The AOPP concentrations were expressed in µmol/L of chloramine-T equivalents.

### 2.6. Determination of SCFAs

The extraction of acetate, propionate, and butyrate was performed as reported by Micalizzi et al. [35]. Briefly, the analysis of the SCFA profiles in human plasma was carried out on a GCMS-QP2020 NX system (Shimadzu, Duisburg, Germany) equipped with an AOC-20i autosampler. The separation of SCFAs was performed on a Nukol 30 m × 0.25 mm ID × 0.25 μm df capillary GC column (Merck Life Science, Darmstadt, Germany). Temp. program: from 100 °C (3 min) to 210 °C at 30.0 °C min^−1^. Helium was used as a carrier gas at a constant linear velocity of 50 cm s^−1^ (initial inlet pressure of 133.0 kPa). The volume injection was 3.0 μL, with a split ratio of 1:5. The MS system acquired the target compounds in selected ion monitoring (SIM) acquisition mode. A quantifier ion (Q) was selected for quantitative purposes, while two qualifier ions (q1 and q2) were utilized to confirm compound identity. The following ions were selected: acetic acid, 60 *m*/*z* (Q), 45 *m*/*z* (q1), and 43 *m*/*z* (q2); propionic acid, 74 *m*/*z* (Q), 73 *m*/*z* (q1), and 57 *m*/*z* (q2); butyric acid, 60 *m*/*z* (Q), 73 *m*/*z* (q1), and 55 *m*/*z* (q2); and 2-ethylbutyric acid (ISTD), 88 *m*/*z* (Q), 73 *m*/*z* (q1), and 55 *m*/*z* (q2). The MS parameters were as follows: ion source temperature 200 °C, and interface temperature 220 °C. The GCMS solution software (version 4.50 Shimadzu) was used for both data acquisition and processing.

### 2.7. Statistical Analysis

The data obtained from the experiments described above are expressed as mean values ± standard deviation of the mean (SD). The Kolmogorov–Smirnov test revealed that the majority of the variables did not follow a normal distribution; so, a statistical analysis was conducted using non-parametric tests.

Spearman’s correlation test was applied to the following results to evaluate the relationship between the variables. The Kruskal–Wallis test and Mann–Whitney U test were used for the comparison of data sets.

Statistical analyses were performed using GraphPad Prism 8 software (San Diego, CA, USA). The differences were considered significant for *p* values < 0.05.

## 3. Results

The demographic and biochemical features of the recruited FM patients are shown in Table 1.

A mean FIQ-R score in the study cohort indicated that the patient presented with moderately severe fibromyalgia symptoms (Table 1). Indeed, five patients (9.6%) could be classified as affected by mild fibromyalgia symptoms (36.44 ± 5.25), twenty-three patients (44.2%) as affected by moderate fibromyalgia symptoms (56.87 ± 12.91), and twenty-four patients (46.1%) as affected by fibromyalgia symptoms of a high degree of severity (76.44 ± 8.27). No patient was classified as being in disease remission. The symptoms most frequently reported by the patients were pain, tiredness, fatigue, stiffness, and reduced quality of sleep.

The vitamin D status in the whole population could be classified as sufficient (Table 1). However, it should be noted that the concentration range varied from 5 to 79 µg/L, with 36.9% of the patients showing insufficient levels, and many patients were relying on vitamin D supplementation.

The assessment of pro-inflammatory cytokines in the FM patients showed that the IL-17 levels were 7 times higher than the upper reference limit, while those of IFN-γ and TNF-α were, respectively, 3.2 and 2.7 times higher than their reference values. On the other hand, the levels of IL-6 and IL-1β were in the normal range. Only 13.23% of the recruited patients had IL-1β concentrations above the reference range (36.39 ± 14.58), while 19.40% of them had elevated IL-6 levels (10.11 ± 3.75).

The measurement of AOPPs showed that the mean AOPP serum levels were 2.4 times higher than the upper reference limit (Table 1), except those in 18.4% of the patients, showing values lower than the reference limit (77.4 ± 11.8).

The Trp levels were slightly within the normal range; however, 28 samples (65.1%) had lower levels than the reference limit (10.23 ± 3.9), while the Kyn contents were in the reference range. In our cohort, the mean values of the Kyn/Trp ratio were 4.86 times higher than the upper reference limits [36,37], and only three results were in the normal range.

Regarding the SCFA content in the FM plasma samples, the acetic acid concentration resulted 2.1 times lower than the minimum physiological range. Instead, the propionic acid and butyric acid concentrations were in the normal range, although 25.42% of the patients showed deficiencies in butyrate content (0.36 ± 0.14). Acetate was the most abundant SCFA, accounting for 89.31% of the total SCFAs, whereas propionate and butyrate accounted for 6.44% and 3.26%, respectively, of the total SCFAs (Table 1).

Initially, we evaluated the impact of age on the variables under consideration, and the statistical analysis revealed no significant correlations.

**Figure 1:**
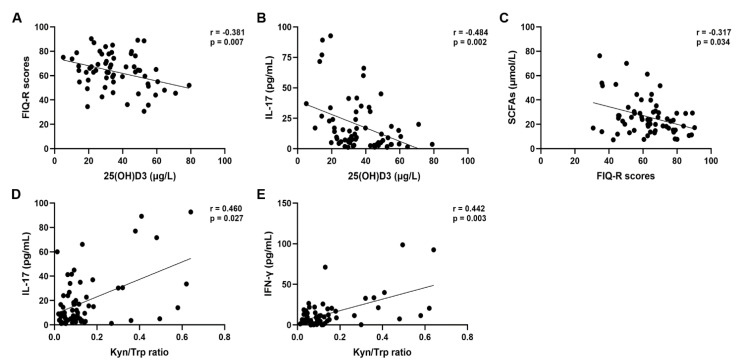
Correlation analysis between 25(OH)D3 serum levels and FIQ-R scores (**A**), 25(OH)D3 serum levels and IL-17 levels (**B**), FIQ-R scores and total SCFA values (**C**), Kyn/Trp ratio and IL-17 levels (**D**), Kyn/Trp ratio and IFN-γ levels (**E**).

Correlation analyses revealed a significantly negative correlation between vitamin D levels and FIQ-R scores (r = −0.381, *p* = 0.007; Figure 1A), particularly, the FIQ-R domain *Symptoms* scores (r = −0.354, *p* = 0.013).

Additionally, significantly negative correlations were found between vitamin D and IL-17 (r = −0.484, *p* = 0.002; Figure 1B) as well as Kyn (r = −0.368, *p* = 0.025).

Moreover, the FIQ-R scores were negatively correlated with acetate (r = −0.328, *p* = 0.028) as well as with the total SCFA values (r= −0.317, *p* = 0.034; Figure 1C). The FIQ-R domain *Symptoms* scores too resulted negatively correlated with the acetate and SCFA values (r = −0.339, *p* = 0.023; r = −0.353, *p* = 0.017, respectively). The FIQ-R domain *Symptoms* scores resulted positively correlated with the Kyn/Trp ratio (r = 0.386, *p* = 0.032).

Regarding the pro-inflammatory cytokines, significantly positive correlations were found between IL-17 and IFN-γ (r = 0.452, *p* = 0.003), as well as TNF-α (r = 0.423, *p* = 0.009), Kyn (r = 0.488, *p* = 0.018) and the Kyn/Trp ratio (r = 0.460, *p* = 0.027; Figure 1D). Similarly, IFN-γ showed a positive correlation with the Kyn/Trp ratio (r = 0.442, *p* = 0.003; Figure 1E) and a negative correlation with the Trp levels (r = −0.321, *p* = 0.036).

Significantly negative correlations were observed between TNF-α and acetate (r= −0.283, *p* = 0.040), as well as propionate (r = −0.367, *p* = 0.007) and total SCFAs (r = −0.334, *p* = 0.015).

Moreover, acetate showed a positive correlation with the propionate levels (r = 0.587, *p* < 0.001), as well as with the total SCFA levels (r = 0.974, *p* < 0.001) and Trp (r = 0.414, *p* = 0.015). Similarly, positive correlations were also found between propionate and butyrate (r = 0.296, *p* = 0.023), as well as total SCFAs (r = 0.668, *p* < 0.001) and Trp levels (r = 0.373, *p* = 0.030). The total SCFA levels were positively correlated with Trp concentrations (r = 0.442, *p* = 0.009).

### 3.1. Clusterization of Patients on the Basis of the FIQ-R Scores

To more deeply understand the relationships between the examined variables and FM impact, we stratified the study cohort in three groups based on the FIQ-R scores, namely, Mild (30.7–45), Moderate (50.3–64.5), and Severe (66–90.3) FM groups (Table 2).

We observed that the vitamin D levels were inversely related to the FIQ-R scores, progressively decreasing while the FIQ-R scores increased, and the lowest concentrations were observed in the Severe FM group (Table 2). Furthermore, significant differences in vitamin D levels were found between the Mild and the Moderate FM groups (*p* = 0.040) and between the Mild and the Severe FM groups (*p* = 0.009).

The concentrations of all pro-inflammatory cytokines, but IL-6, were higher in the Moderate and Severe FM groups than in the Mild FM patients, although the differences were not statistically significant. IL-1β and IL-6 showed values within or near the normal reference range, in contrast to the cytokines IL-17, IFN-γ, and TNF-α, which showed concentrations above the physiological cut-off in all three groups (Table 2).

Similarly, AOPPs and the Kyn/Trp ratio revealed the highest values in the Severe FM group.

Total SCFAs, expressed as the sum of acetate, propionate, and butyrate, showed the highest concentrations in the Mild FM group. Notably, acetate and propionate, but not butyrate, showed the lowest concentrations in the Severe FM group (Table 2).

### 3.2. Clusterization of Patients on the Basis of the Vitamin D Status

A further stratification of our study cohort was carried out based on the vitamin D status, resulting in two groups of patients, namely, Vitamin D-Insufficient (5–29.9 μg/L) and Vitamin D-Sufficient (30.4–79 μg/L) (Table 3).

The lowest FIQ-R scores, indicating a classification of the patients in the Moderate FM group, were found in the Vitamin D-Sufficient group and were significantly different from the FIQ-R scores of the FM patients in the Vitamin D-Insufficient group (*p* = 0.017). Notably, the patients in both groups showed similar scores in the different FIQ-R domains except for the *Physical function* domain, whose score resulted significantly higher in the Vitamin D-Insufficient group (*p* = 0.030) (Table 3).

Similarly, the lowest levels of pro-inflammatory cytokines were found in the Vitamin D-Sufficient group, except for TNF-α, although the differences with the Vitamin D-Insufficient group were not significant. In contrast, a significant difference between the two groups was observed for the IL-17 levels (*p* = 0.018) (Table 3). The concentrations of IL-1β and IL-6 were within the normal reference range, while those of IL-17, IFN-γ, and TNF-α were above the cut-off in both groups.

The AOPP levels were higher than the normal reference limit in both groups, with the highest concentrations in the FM patients of the Vitamin D-Insufficient group.

A similar trend was observed for the Kyn/Trp ratio, which showed the highest values in the Vitamin D-Insufficient group, with a significant difference compared to the Vitamin D-Sufficient group for the Kyn levels (*p* = 0.045) (Table 3).

The levels of total SCFAs were higher in the Vitamin D-Sufficient group, primarily due to an increase in acetate levels.

To better understand how the vitamin D status interacts with other clinical variables, we performed correlation analyses separately on Vitamin D-Insufficient and Vitamin D-Sufficient patients.

The data analyses for the Vitamin D-Insufficient group showed a significantly positive correlation between the *Physical function* domain score and the AOPP levels (r = 0.850, *p* = 0.006). Moreover, the FIQ-R domain *Symptoms* scores were negatively correlated with the total SCFA values and Trp levels (r = −0.512, *p* = 0.038; r = −0.655, *p* = 0.034, respectively). Regarding the pro-inflammatory cytokines, in this group, significantly negative correlations were found between TNF-α and acetate (r = −0.525, *p* = 0.021), as well as total SCFAs (r = −0.542, *p* = 0.016).

Correlation analyses in the Vitamin D-Sufficient group revealed a significantly negative correlation between the vitamin D levels and the FIQ-R scores (r = −0.369, *p* = 0.04). Additionally, the *Physical function* domain score was negatively correlated with acetate and total SCFAs (r = -0.465, *p* = 0.017; r = −0.505, *p* = 0.009, respectively), The levels of propionate were negatively correlated with the TNF-α levels (r = −0.490, *p* = 0.006) and positively correlated with the Trp levels (r = 0.510, *p* = 0.018).

## 4. Discussion

Chronic pain affects patients’ functioning and quality of life and is increasingly recognized as a relevant social burden due to its impact on health care utilization and productivity. Mounting evidence indicates that chronic pain is elicited by a decrease in the pain threshold and an increase in the pain response triggered by an excess of pro-inflammatory mediators released by infiltrating or resident immune cells and glia that activate or sensitize peripheral nociceptors [1,17,18,38,39,40,41,42,43].

In fibromyalgia, central and peripheral sensitization leads to musculoskeletal symptoms and a wide range of extra-skeletal disorders affecting various organs. At the same time, comorbidities are very common, particularly autoimmune diseases, such as Hashimoto’s thyroiditis, Sjögren’s syndrome, rheumatoid arthritis, multiple sclerosis, and others [44]. The lack of specific biomarkers delays diagnosis and complicates the patient’s condition.

Recent findings indicate that hypovitaminosis D, which is widespread in the Mediterranean area despite good solar radiation, is associated with the up-regulation of inflammatory markers also in the general healthy population [45,46,47,48].

Conflicting observations have been reported about the role of vitamin D in FM, with few studies describing lower vitamin D levels in FM patients than in healthy subjects and a negative correlation between vitamin D and FM severity [32,49,50,51,52], and others showing not-significant differences between patients and controls [53,54].

In our study cohort, only 36.9% of the FM patients had insufficient vitamin D levels, while the remaining had sufficient levels. Notably, a deeper anamnestic evaluation revealed that many patients, who were not newly diagnosed and had long-standing conditions, were taking vitamin D supplements to manage their symptoms.

Interestingly, a significantly positive correlation was found between vitamin D levels and FIQ-R scores as well as FIQ-R *Symptoms* domain scores for the recruited women. This suggests that the vitamin D levels may play a role in modulating chronic pain associated with fibromyalgia, in line with the available literature data [49,53,55,56]. Similar conclusions can be drawn when examining variables in groups divided by FIQ-R scores and vitamin D status. The women with higher FIQ-R scores, indicating moderate to severe FM, had the lowest average vitamin D levels, with significant differences compared to those experiencing lower pain intensity.

Interestingly, correlation analyses performed separately in the two groups of patients clustered together according to either vitamin D sufficiency or vitamin D insufficiency showed different correlations between the examined metabolic features. In particular, in the Vitamin D-Insufficient group of patients, physical functions resulted compromised by oxidative stress and the severity of symptoms, and the corresponding score was correlated with a reduction in the SCFA and Trp levels that was associated with increased inflammation. In the Vitamin D-Sufficient group of patients, disease severity was in general attenuated by high vitamin D levels, and physical functions were strongly influenced by SCFAs, whose levels were negatively correlated with the inflammation levels.

Vitamin D plays a key role in regulating the expression of many antioxidant and pro-inflammatory systems. For instance, it controls the expression of nuclear factor-erythroid-2-related factor 2 (Nrf2), a redox-sensitive transcription factor that activates numerous genes involved in antioxidant and detoxifying enzyme production [28]. Additionally, vitamin D provides protection against oxidative stress in the nervous system, since it attenuates the inflammatory responses through immunomodulation and contributes to the synthesis and release of neurotransmitters, such as dopamine, gamma-aminobutyric acid, and serotonin [57,58]. These neurotransmitters are known to be dysregulated in fibromyalgia, which provides further evidence of their association with chronic pain.

In this context, it becomes essential to assess the involvement of immune system cells, specifically Th1 and Th17, along with the pro-inflammatory cytokines that are produced by these cell types and the resulting oxidative stress condition. Elevated serum cytokine concentrations have been identified in several studies conducted on patients with complex regional pain, peripheral neuropathy, neuropathic pain [59,60,61], and also fibromyalgia [62,63,64].

In our study cohort, the pro-inflammatory cytokines IL-17, IFN-γ, and TNF-α, along with AOPPs as markers of oxidative stress, showed levels above the normal reference limits, while only 16% of the patients had IL-1β and IL-6 concentrations above the physiological range. The positive correlations between IL-17, IFN-γ, and TNF-α demonstrated a unique pattern. Notably, the high levels of AOPPs and all cytokines, but IL-6, in the Moderate and Severe FM groups indicated a more pronounced oxidative and inflammatory stress and a greater lymphocyte activation in the advanced stages of the disease, although the differences with respect to the Mild FM group were not significant.

A significantly negative correlation was observed between IL-17 and vitamin D, highlighting the anti-inflammatory role of vitamin D. It is remarkable that, although all cytokines, except for TNF-α, and AOPPs showed higher levels in the Vitamin D-Insufficient group than in the Vitamin D-Sufficient group of FM patients, significant differences were observed only for IL-17, which is a marker of Th17 lymphocyte activation.

The expansion of Th17 lymphocytes plays a crucial role in chronic pain, autoimmune diseases, IBD, and fibromyalgia [65,66,67,68,69,70]. An analysis of the Th17 and IL-17 levels in peripheral blood revealed a significant increase in T lymphocytes in an herniated disc group compared to healthy controls, suggesting that alterations in Th17 lymphocytes may contribute to the pain experienced by these patients [65]. Here, we showed an increase in IL-17 and a positive correlation with other cytokines such as TNF-α and IFN-γ, confirming previous observations in FM patients [5,52], and also an increase in IL-17 with increasing FIQ-R scores. Given the negative correlation between IL-17 and vitamin D, we can hypothesize that vitamin D contributes to the attenuation of FM severity by exerting immunomodulatory effects on Th17 lymphocytes. This hypothesis is further corroborated by the negative correlation between Vitamin D levels an Kyn concentrations. Indeed, the Kyn levels and the Kyn/Trp ratio were significantly lower in the Vitamin D-Sufficient group than in the Vitamin D-Insufficient one. Notably, the FIQ-R *Symptoms* domain score was positively correlated with the Kyn/Trp ratio, which was markedly higher than the reference limits, suggesting that this ratio has a greater impact on FM pain intensity and symptoms than other variables. Furthermore, the Kyn/Trp ratio was positively correlated with IL-17 and IFN-γ.

Given these results, the Kyn/Trp ratio may have a role in the different types of fibromyalgia symptoms because it exhibits a pattern resembling that of pro-inflammatory cytokines. A more detailed analysis, carried out by dividing the population into groups, revealed that Kyn and the Kyn/Trp ratio showed higher average values in the Moderate and Severe FM groups, although the differences compared to the Mild FM group were not statistically significant.

Only the study by Golnaz Barjandi and collaborators investigated the involvement of this variable in fibromyalgia and temporomandibular disorder, but did not correlate it with any biochemical markers [71].

The Kyn/Trp ratio can be considered a surrogate marker for IDO1 activity, as it increases in response to elevated kynurenine production. IDO-1 is an enzyme whose activity and expression are highly stimulated by pro-inflammatory cytokines produced by Th1 and Th17 lymphocytes, such as IFN-γ [72]. The IDO1/kynurenine pathway has been studied in various chronic inflammatory diseases, including arthritis, where overexpression of the enzyme has been observed [73,74], as well as in psychological disorders such as depression [75]. Therefore, in fibromyalgia, lymphocyte activation leads to increased production of pro-inflammatory cytokines and heightened IDO1 activity. This results in a shift in Trp metabolism towards Kyn production, an increase in the Kyn/Trp ratio, and an amplification of chronic inflammatory pain. Moreover, sufficient levels of vitamin D may be helpful to reduce inflammation and pain.

Many bacterial species, whose abundance is reduced in fibromyalgia patients, play a crucial role in the metabolism of short-chain fatty acids (SCFAs). Research on fecal samples has revealed that FM patients have lower levels of certain SCFAs than controls, underscoring the critical function of a specific diet [22,76].

In our study, we assessed the plasma concentration of SCFAs in FM patients. It is known that acetate is predominant among the circulating SCFAs in humans [77], while the plasma levels of other SCFAs are significantly reduced compared to their stool concentrations. In our population, despite accounting for approximately 89% of the total SCFAs, acetate concentration resulted deficient, while propionate and butyrate concentration was in the normal reference range. Interestingly, acetate concentration decreased in patients with Moderate and Severe FM compared to those with Mild FM. Moreover, a moderate increase in acetate concentration was observed in FM patients with sufficient vitamin D levels compared to those with insufficient levels. Although these variations were not statistically significant, a correlation analysis revealed important significant relationships between the variables. Notably, the total FIQ-R score, along with the FIQ-R *Symptoms domain* score, showed negative correlations with total SCFAs and acetate. Significant negative correlations were observed between TNF-α and acetate, as well as propionate and total SCFAs. It is also worth noting the positive correlation between tryptophan levels and acetate, propionate, and total SCFAs.

SCFAs play a crucial role in immunomodulation, counteracting the pro-inflammatory effects of certain cytokines on the intestinal epithelium [78] and interacting with neutrophils and T and B lymphocytes to either stimulate or suppress their local activity [79]. Therefore, a deficiency in short-chain fatty acids also leads to the suppression of immune tolerance, resulting in increased inflammation. Our study highlights that the pro-inflammatory interleukins IL-17, IFN-γ, and TNF-α follow an opposite trend to that of SCFAs, particularly when the population is stratified based on the FIQ-R score. This finding is important as it not only confirms a connection between SCFA concentrations and the activity of the immune system in FM patients, but also suggests that dysbiosis, driven by a deficiency in these fatty acids, may be linked to higher FIQ-R scores, elevated Kyn/Trp ratios, and lower vitamin D levels.

It is worth noting the importance of SCFAs in modulating the permeability of the blood–brain barrier through tight junctions [80]. In cases of SCFA depletion, LPS, pro-inflammatory cytokines, activated immune cells, and microbial metabolites (such as Kyn and QA) could enter the central nervous system, leading to inflammation.

Vitamin D not only plays a role in managing chronic pain but also helps maintain the integrity of tight junctions. Its deficiency, as demonstrated in experimental studies, disrupts tight junction homeostasis, leads to intestinal barrier dysfunction and mucosal damage, and increases the susceptibility to infections [81,82,83]. The dysbiosis associated with vitamin D deficiency can be reversed when optimal vitamin levels are restored: oral supplementation of vitamin D3 has been shown to alter the composition of the gut microbiota by reducing opportunistic pathogens and promoting bacterial diversity [84].

These findings highlight the additional benefit of vitamin D supplementation in managing dysbiosis and chronic inflammatory pain associated with fibromyalgia.

The limitations of this study are mainly represented by the small number of recruited patients, the lack of a healthy control group, and the presence of potentially confounding factors, such as the use of vitamin D supplements by most of the recruited patients and the use of pain management medications, like opioids. Indeed, the size of the study cohort was affected by the low prevalence (2–3%) of FM [2] in the general population, so that a long time for the recruitment of a large number of study subjects would be required. The issue of vitamin D supplementation cannot be easily addressed, since almost all FM patients are used to take vitamin D supplements. The only way for overcoming this limitation could have been the recruitment of newly diagnosed, treatment-naïve patients, who are hard to find in a reasonable time because of the already mentioned low FM prevalence in the general population. Additionally, comorbidities, including other chronic pain conditions, are common in patients with FM and can further complicate the clinical picture, being also a confounding factor for the characterization of pathogenic mechanisms and the identification of new specific diagnostic markers. Furthermore, acute inflammatory diseases, chronic inflammatory bowel disease, metabolic disorders, diabetes mellitus, and celiac disease could represent confounding factors in gut microbiota analyses. However, even if the study was primarily aimed to understand whether differences in the vitamin D status are able to affect the severity of the inflammatory status and disease symptoms, we must highlight that the inclusion of a healthy control group would have strengthened the study validity, so that the present findings should be considered cautiously.

## 5. Conclusions

Our preliminary data demonstrate, for the first time, that the plasma SCFA levels, along with the Kyn/Trp ratio, are altered in patients with fibromyalgia, suggesting the presence of dysbiosis, in line with previously reported findings by other researchers. Thus, not only insufficient vitamin D levels but also low SCFA concentrations and an elevated Kyn/Trp ratio are associated with more severe FIQ-R scores and higher concentrations of pro-inflammatory cytokines. The positive correlation found between the Kyn/Trp ratio and the levels of the IL-17 and IFN-γ cytokines may suggest that the Kyn/Trp ratio holds the potential to represent a surrogate marker of lymphocyte activation, but further investigations are needed to confirm its usefulness as an alternative diagnostic biomarker.

In conclusion, a larger study cohort, the use of more stringent exclusion criteria, and a case–control methodological approach are required to confirm our hypotheses and provide additional insights.

## Figures and Tables

**Table 1:** Demographic and biochemical features of the FM population.

Variables	Mean ± SD (Range)	Reference Values
Age (years)	49.9 ± 12.35 (16–75)	/
FIQ-R scores	64.76 ± 13.67 (30.7–90.3)	≤30 remission >30 and ≤45 mild >45 and ≤65 moderate >65 severe
1. *Physical function*	19.17 ± 4.76 (9–28.7)	0–30
2. *General health*	12.64 ± 3.99 (4–19)	0–20
3. *Symptoms*	33.96 ± 7.74 (10–45.5)	0–50
25(OH) vitamin D3 (μg/L)	35.80 ± 15.4 (5–79)	<30 insufficient >30 sufficient
IL-1β (pg/mL)	10.14 ± 11.98 (1.0–64.77)	<15
IL-6 (pg/mL)	4.30 ± 3.46 (0.98–16.95)	<6
IL-17 (pg/mL)	20.9 ± 23.97 (1.2–92.7)	<3
IFN-γ (pg/mL)	13.53 ± 18.71 (0.1–98.7)	<4.2
TNF-α (pg/mL)	21.81 ± 16.73 (1.09–82.26)	<8.1
AOPP (μM)	240.75 ± 217.59 (62.49–1235.21)	<100
Trp (μmol/L)	15.13 ± 9.42 (3.2–48.27)	15–59
Kyn (μmol/L)	1.41 ± 0.84 (0.22–5.19)	0.92–2.68
Kyn/Trp ratio	0.13 ± 0.14 (0.013–0.64)	<0.0267
SCFAs (μmol/L)	26.11 ± 15.55 (7.32–76.3)	51–120
- *Acetate*	23.86 ± 15.19 (5.3–72.82)	50–100
- *Propionate*	1.39 ± 0.70 (0.62–4.28)	0.5–10
- *Butyrate*	0.85 ± 0.86 (0.07–5.23)	0.5–10

Data are expressed as mean ± SD.

**Table 2:** Variability of the biomarkers examined in the FM population divided into subgroups on the basis of the FIQ-R scores.

Variables	Mild	Moderate	Severe	Reference Values
FIQ-R scores	36.44 ± 5.25	56.87 ± 12.91	76.44 ± 8.27	
1. *Physical function*	14.74 ± 4.93	16.18 ± 3.95	22.66 ± 3.54	0–30
2. *General health*	7.66 ± 2.08	10.06 ± 3.82	15.34 ± 2.63	0–20
3. *Symptoms*	17.1 ± 5.06	31.0 ± 7.82	38.43 ± 4.19	0–50
Age (years)	50.8 ± 14.41	49.56 ± 14.78	50 ± 9.87	/
25(OH) vitamin D3 (μg/L)	48.14 ± 18.96	33.98 ± 16.21 *	29.24 ± 9.26 **	<30 insufficient >30 sufficient
IL-1β (pg/mL)	7.58 ± 3.02	12.74 ± 14.32	9.34 ± 12.11	<15
IL-6 (pg/mL)	6.40 ± 2.51	3.95 ± 2.73	3.89 ± 3.05	<6
IL-17 (pg/mL)	10.67 ± 9.89	17.29 ± 20.79	22.50 ± 23.10	<3
IFN-γ (pg/mL)	7.56 ± 5.03	9.17 ± 8.80	17.28 ± 24.66	<4.2
TNF-α (pg/mL)	17.97 ± 9.40	22.18 ± 20.29	21.17 ± 21.08	<8.1
AOPP (μM)	157.79 ± 70.97	210.17 ± 126.01	337.8 ± 340.5	<100
Trp (μmol/L)	12.33 ± 2.93	15.24 ± 10.11	16.57 ± 11.40	15–59
Kyn (μmol/L)	0.69 ± 0.45	1.49 ± 1.21	1.37 ± 0.55	0.92–2.68
Kyn/Trp ratio	0.05 ± 0.02	0.12 ± 0.13	0.13 ± 0.12	<0.0267
SCFAs (μmol/L)	37.22 ± 26.40	26.86 ± 11.63	23.23 ± 11.69	51–120
- *Acetate*	35.27 ± 25.53	24.62 ± 11.20	20.93 ± 11.49	50–100
- *Propionate*	1.50 ± 1.10	1.35 ± 0.52	1.32 ± 0.56	0.5–10
- *Butyrate*	0.44 ± 0.22	0.89 ± 0.81	0.98 ± 1.14	0.5–10

Data are expressed as mean ± SD. * *p* < 0.05 and ** *p* < 0.01 in comparison with mild fibromyalgia.

**Table 3:** Variability in the biomarkers examined in the FM population divided into subgroups on the basis of the vitamin D status.

Variables	Insufficient	Sufficient	Reference Values
25(OH)vitaminD3 (μg/L)	20.73 ± 6.53	44.74 ± 11.88	
Age (years)	48.81 ± 13.50	50.55 ± 11.96	/
FIQ-R scores	68.65 ± 13.44	61.30 ± 12.79 *	≤30 remission >30 and ≤45 mild >45 and ≤65 moderate >65 severe
1. *Physical function*	20.29 ± 5.49	18.17 ± 3.95 *	0–30
2. *General health*	13.04 ± 4.03	12.10 ± 3.88	0–20
3. *Symptoms*	35.33 ± 7.46	32.56 ± 7.64	0–50
IL-1β (pg/mL)	11.43 ± 12.69	9.25 ± 11.19	<15
IL-6 (pg/mL)	4.89 ± 4.34	3.67 ± 2.70	<6
IL-17 (pg/mL)	29.77 ± 28.25	12.93 ± 15.16 *	<3
IFN-γ (pg/mL)	15.09 ± 21.18	10.98 ± 13.27	<4.2
TNF-α (pg/mL)	19.57 ± 18.56	22.44 ± 13.26	<8.1
AOPP (μM)	341.08 ± 355.34	223.21 ± 134.91	<100
Trp (μmol/L)	16.91 ± 8.54	16.15 ± 9.75	15–59
Kyn (μmol/L)	1.78 ± 1.17	1.15 ± 0.61 *	0.92–2.68
Kyn/Trp ratio	0.15 ± 0.16	0.08 ± 0.04	<0.0267
SCFAs (μmol/L)	25.35 ± 15.10	26.85 ± 13.74	51–120
- *Acetate*	22.83 ± 14.50	24.79 ± 13.50	50–100
- *Propionate*	1.34 ± 0.60	1.34 ± 0.50	0.5–10
- *Butyrate*	1.17 ± 1.15	0.71 ± 0.67	0.5–10

Data are expressed as mean ± SD. * *p* < 0.05 in comparison with Vitamin D-Insufficient group.

## Data Availability

The original contributions presented in this study are included in the article. Further inquiries can be directed to the corresponding author.
